# Cytotoxicity, Phytochemical, Antiparasitic Screening, and Antioxidant Activities of *Mucuna pruriens* (Fabaceae)

**DOI:** 10.3390/plants9091249

**Published:** 2020-09-22

**Authors:** Mahboob Adekilekun Jimoh, Oladayo Amed Idris, Muhali Olaide Jimoh

**Affiliations:** 1 Department of Plant Biology, Osun State University, Osogbo P.M.B. 4494, Nigeria; mahboob.jimoh@uniosun.edu.ng; 2 Department of Botany, University of Fort Hare, Alice 5700, South Africa; dayoamed@gmail.com; 3 Unit for Environmental Sciences and Management (UESM), Faculty of Natural and Agricultural Sciences, North-West University, Potchefstroom 2531, South Africa; 4 Department of Horticultural Sciences, Cape Peninsula University of Technology, Bellville 7537, South Africa

**Keywords:** antiparasitic, human cervix adenocarcinoma, *Mucuna pruriens*, pharmaceutical precursors, phenolic acids

## Abstract

This study aimed at assessing the biological activities of *Mucuna pruriens* seeds using cytotoxicity, phytochemical, antiparasitic screening, and antioxidant assays. Mature fruits of *M*. *pruriens* were harvested from Fort Hare University’s Research Farm located in Alice, South Africa. The collected seeds were pulverized in a standard process and taken to the laboratory for crude extraction and further treatments. Cytotoxic, antimalarial, and trypanocidal effects of crude extracts obtained from ethanol and water were tested, while the total phenolic, proanthocyanidin, and flavonoid contents of the aqueous extracts as well as their pharmacological activities were determined in vitro using 2,2-diphenyl-1-picrylhydrazyl ethanol (DPPH), ferric reducing antioxidant power (FRAP), and 2,2′-azino-bis(3-ethylbenzothiazoline-6-sulphonic acid) (ABTS) assays. Although the extracts showed mild antiparasitic (antiplasmodial and trypanocidal) effects, results from the cytotoxic experiment revealed that *M*. *pruriens* is not toxic to human cervix adenocarcinoma (HeLa) cells when tested using 50 µg/mL of extracts. It was observed that the seeds were remarkably rich in phenol (3730.1 ± 15.52 mg gallic acid equivalent (GAE)/g) compared to flavonoids (63.03 ± 1.95 mg quercetin equivalent (QE)/g) and proanthocyanidin (18.92 ± 1.09 mg catechin equivalent (CE)/g). Also, the antioxidant activities of the extracts were comparable to those of the standard antioxidant drugs (rutin and gallic acid) used, in a concentration-dependent manner. There was a direct relationship between phenolic acid content and antioxidant effects. It is therefore suggested that *M. pruriens* seeds be incorporated into human diets as a supplement to promote healthy living. Pharmaceutical industries with a particular interest in natural phenolic acids should consider using seeds of *M. pruriens* as pharmaceutical precursors.

## 1. Introduction

*Mucuna pruriens* (Fabaceae) is native to tropical climate, particularly India, Africa, and some parts of China [1]. It is an annual climbing legume with fleshy pods containing about six ellipsoid seeds [2]. *M*. *pruriens* is a valuable medicinal plant used to treat several diseases like malaria, cancer, epilepsy, Parkinson’s disease, diarrhea, helminthiasis, ulcer, infertility, snakebite, scorpion stings, and elephantiasis [1,3,4,5]. The plant serves as a cover crop usually cultivated as effective green manure to replenish devastated soil due to its ability to accumulate nutrients in various environments [6,7]. It exhibits allelopathy to weed growth and is efficient in reducing the nematode population in farms [1,8].

Several studies have shown that L-3,4-dihydroxyphenylalanine (L-DOPA), lectin, isoflavanones, and some alkaloids contained in *M*. *pruriens* seeds are responsible for the enormous bioactivity of its crude extracts [9,10]. Recently, several therapeutic approaches have been adopted to make healthy living affordable to all regardless of their status in the society, since synthetic products are not only expensive but also come with adverse effects [11,12]. The challenges posed by the cost and safety of synthetic drugs have raised the need to explore underutilized plant species reputed for high medicinal significance [12,13,14]. From previous reports, different parts of *M*. *pruriens* have proven to be good for diverse therapeutic purposes [5,6]. Despite the outcome of these novel researches, plants like *M*. *pruriens* with significant biological activities have not been adequately exploited for their therapeutic potential.

Besides multi-drug resistance to synthetic drugs found in some communities, which is a major setback in the fight against malaria and other chronic diseases, the long-term usage of synthetic drugs could be detrimental. Countering the predisposing factors to these phenomena is a major concern for pharmaceutical industries. Some of these drugs are not readily available, not affordable, or could be imbued with myriads of adverse effects upon usage [15,16], thereby causing an ethical and medical dilemma. It has therefore become expedient to explore the medicinal efficacy of some medicinal plants such as *M*. *pruriens* to downplay the dilemma created by synthetic drugs, decrease the possibility of drug resistance, increase drug efficiency, and provide affordable medicinal remedies [17].

To contribute to the increasing demand for plant-based products as natural remedies, this study seeks to assess the cytotoxic, antimalarial, and trypanocidal potency of *M*. *pruriens* used in folk medicine and then evaluate the phytochemicals and antioxidant activities of the plant. It is our informed opinion that the outcome of this research will assist pharmaceutical industries and households that may want to explore the plant as a food supplement or for medicinal purposes.

## 2. Materials and Methods

### 2.1. Plant Materials

Mature fruits of *M*. *pruriens* were harvested from Fort Hare University’s Research Farm located in Alice, South Africa. Fresh seeds collected from the pods were air-dried, pulverized, and stored in air-tight containers kept in a refrigerator set at 4 °C for further laboratory treatment. 

### 2.2. Crude Extraction

Ethanol of standard analytical grade and distilled water were used for extraction. The two solvents were selected due to the well-known pharmacological effects of ethanol and food value of water. More so, water and ethanol are reported to be common extractants of *M*. *pruriens* bioactive compounds [1]. About 25 g of the pulverized seeds of *M*. *pruriens* was transferred in conical flasks filled with 500 mL each of water and ethanol. The conical flasks and their contents were shaken in an orbital incubator shaker (Gallenkamp & Co. Ltd., London, England) for 48 h. Thereafter, the crude extracts were filtered through a Whatman No. 1 filter paper placed in a Buchner funnel connected to an electric vacuum pump. The aqueous filtrate obtained was chilled in a refrigerant set at −40 °C (Poly-Science AD15R-40-A12E-USA; Serial No: 1C1490106) and concentrated to dryness with a freeze dryer (Savant vapour trap, RV-T41404, San Diego, CA, USA) for 24 h. A rotary evaporator (LABOROTA 4000 Heidolph, Schwabach, Germany; Serial No: 090409460) set at 78 °C was also used to concentrate the ethanolic filtrate to dryness. The prepared extracts were then stowed in sterile bottles kept at 4 °C in a refrigerator until further use. 

### 2.3. Cytotoxicity Assay

HeLa cells were maintained in Dulbecco’s Modified Eagle’s Medium (DMEM) with 5 mM of L-glutamine (Lonza) and then fortified with 10% fetal bovine serum (FBS). The cells were incubated at 37 °C in a 96-well plate at a density of 5 × 10^3^ cells per well for 24 h in a 5% CO_2_ humidified chamber. The cytotoxicity of the extract was assessed by subsequently exposing the HeLa cells to 50 µg/mL of crude extracts at a fixed concentration in the 96-well microplate for 48 h. The number of cells that survived the drug treatment was determined using the resazurin-based reagent and by reading resorufin fluorescence in a Spectramax M3 microplate reader (Molecular Devices LLC, San Jose, CA, USA) The results were expressed as percentage viability in resorufin fluorescence-treated extracts per well, using the untreated wells as controls. The cytotoxic effect of the plant extracts was measured in duplicates, and emetine was used as the positive control for reference purposes.

### 2.4. Antimalarial Assay

The antimalaria experiment was performed as described [16]. In this assay, malaria parasites (*Plasmodium falciparum* strain 3D7) were preserved in RPMI 1640 medium containing 25 mM Hepes and 2 mM L-glutamine (Lonza). About 5% Albumax II, 20 mM glucose, 0.65 mM hypoxanthine, 60 µg/mL gentamycin, and 2–4% hematocrit human red blood cells were added to the medium. Thereafter, the parasites were cultured at 37 °C under an atmosphere of 90% N_2,_ 5% O_2_, and 5% CO_2_ in a closed T75 culture flask. Then, 25 µg/mL of crude extracts of *M. pruriens* was combined with the parasite cultures prepared in a 96-well microplate and incubated in a CO_2_ incubator maintained at 37 °C for 48 h. Thereafter, 20 µL of the incubated mixture was taken from each well and added to 125 µL of Malstat and nitroblue tetrazolium (NBT)/ phenazine ethosulfate (PES) solutions mixed in a new microplate. The activity of the parasite lactate dehydrogenase (pLDH) enzyme component of the cultures was measured in Malstat and NBT/PES solutions. The formation of a purple product revealed by a Spectramax M3 microplate reader at 620 nm indicated the presence of pLDH and, thus, the number of parasites present.

For each sample concentration, pLDH activity in sample-treated wells relative to untreated controls was estimated as percentage parasite viability. Samples were examined in duplicate, and standard deviations (SD) were calculated. Also, the standard anti-malarial drug chloroquine (IC_50_ range = 0.01–0.05 µM) was used for comparative purposes. IC_50_ is the concentration of crude extracts capable of causing 50 % inhibition of the parasite’s growth. 

### 2.5. Trypanocidal Assay

To assess trypanocidal activity as described by [16], a fixed concentration of 25 µg/mL of crude extracts was mixed with cultures of *Trypanosoma brucei* in a 96-well plate, and the mixtures were incubated for 48 h. After incubation, the parasites that survived the treatment with the tested samples were enumerated by the addition of a resazurin-based reagent. Cells were considered viable when they were capable of reducing resazurin to resorufin (a fluorophore with Exc_560_/Em_590_). Results were expressed as percentage parasite viability of the resorufin fluorescence in compound-treated wells relative to untreated controls (pentamidine), determined by a Spectramax M3 microplate reader (Molecular Devices, CA, USA). Compounds were tested in duplicate, and standard deviations (SD) were evaluated. Extracts that reduced parasite viability to <20% were considered for further testing (dose–response cytotoxicity assays). Pentamidine is a common drug used for the treatment of trypanosomiasis.

### 2.6. Phytochemical Screening

#### 2.6.1. Total Phenol

The total phenolic content of the aqueous extract was evaluated using the Folin–Ciocalteu’s technique described in [18], with minor modifications. Gallic acid (standard) was prepared in methanol at an initial concentration of 1.0 mg/mL. This was later diluted and graded in a series of 1.0–0.2 mg/mL, prepared in separate test tubes. The crude extract was also prepared in methanol at a concentration of 1.0 mg/mL. To every 0.5 mL of graded concentration of standard solutions (0.2–1.0 mg/mL), 2.5 mL of Folin–Ciocalteu solution was added and, subsequently, 2 mL of anhydrous Na_2_CO_3_ (7.5% *w*/*v*). The mixture was vortexed and incubated in a water bath set at 40 °C for 30 min; after incubation, the absorbance of the solution was measured at 765 nm using a UV-3000 PC spectrophotometer. The total phenolic component was estimated as milligram of gallic acid equivalent (GAE) per gram of crude extract extrapolated from the standard equation y = 0.1197x − 0.0008 (R^2^ = 0.9991) by the equation $\frac{CV}{M}$ given above, where C is the concentration deduced from the standard calibration graph, V is the volume of plant sample in mL, and M is the mass of the plant sample used, measured in g.

#### 2.6.2. Total Flavonoids 

The total flavonoid component was measured following the aluminum chloride spectrophotometric assay described by [19], with slight modifications. The assay is based on the estimation of the yellow-orange color resulting from the reaction between AlCl_3_ and flavonoids. The aqueous extract and standard (quercetin) stocks were prepared in methanol at a ratio of 1:1. The standard solution was graded in a series of 0.2–1 mg/mL of quercetin in methanol. Thereafter, plant extract (0.5 mL) and graded standard solution (0.5 mL) were pipetted into separate test tubes. Later, distilled water (2 mL) was added, followed by 5% NaNO_2_ (0.15 mL). The mixture was vortexed and left for 6 min. After 5 min, the mixture was supplemented with 10% AlCl_3_ (0.15 mL), followed by 1 M NaOH (1 mL). The solution was brought to 5 mL with distilled water, and the resulting mixture was incubated in a water bath set at 40 °C for 20 min. The solution’s absorbance was measured at 430 nm, and total flavonoid content was evaluated as mg of quercetin equivalent per gram of the extract (QE/g), evaluated from the standard curve y = 1.1829x + 0.0399 (R^2^ = 0.9929) by the equation $\frac{CV}{M}$. C is the concentration evaluated from the standard linear graph equation, V is the volume of extract in mL, and M is the mass of the extract used in g.

#### 2.6.3. Proanthocyanidin Content (Condensed Tannin) 

This was estimated according to [20] with slight modifications. About 0.5 mL each of the tested samples (plant extracts and graded standard solution) was mixed with vanillin (3 mL, 4% *w*/*v*), and later HCl (1.5 mL) was added. After vortexing, the resultant solution was incubated for 15 min at room temperature. Each sample was prepared in three replicates and measured at an absorbance of 500 nm by a spectrophotometer. The total proanthocyanidin content was estimated as mg of catechin equivalent (CE/g) per gram of crude extract, derived from the equation y = 0.5631x − 0.0279 (R^2^ = 0.9518) estimated from the relationship $\frac{CV}{M}$.

### 2.7. Antioxidant Assays

#### 2.7.1. Free Radical Scavenging Activity Using 2,2-Diphenyl-1-picrylhydrazyl (DPPH) 

The free radical scavenging capacity of the test sample was estimated as described by [21], with slight modifications. A solution of 0.135 mM DPPH in methanol was prepared in a dark bottle. The DPPH solution was reacted with graded concentrations (0.04, 0.02, 0.01, 0.005, 0.0025 mg/mL) of the aqueous extract and rutin in a ratio of 1:1. The resulting mixture was vortexed, incubated at room temperature for 30 min, and measured at an absorbance of 517 nm. The scavenging activity of the tested samples was estimated by the equation (Equation (1)):

(1) $$\%\;\mathrm{scavenging}\;\mathrm{activity}\;\mathrm{of}\;\mathrm{DPPH}\;=\;[\frac{\left(\mathrm{Abs}\;\mathrm{control}\;-\;\mathrm{Abs}\;\mathrm{sample}\right)}{\left(\mathrm{Abs}\;\mathrm{control}\right)}]\;\times \;100$$

#### 2.7.2. Ferric Reducing Antioxidant Power (FRAP)

This was assessed following [17], with some modifications. The FRAP reagent was constituted from a solution of 2.5 mL of 0.2 M phosphate buffer prepared from a mixture of 62.5% monobasic dihydrogen phosphate and 37.5% dibasic monohydrogen phosphate (pH 6.6) with 2.5 mL of K_3_Fe(CN)_6_ (potassium hexacyanoferrate). The freshly prepared FRAP reagent was added to serially graded concentrations of aqueous extract and standard drugs in a series of 0.4, 0.2, 0.1, 0.05, and 0.025 mg/mL. The resulting mixture was incubated for 20 min in a water bath set at 50 °C. Thereafter, 2.5 mL of 10% trichloroacetic acid (*w*/*v*) was added to the mixture which was later centrifuged for 10 min at 3000 rpm. The reducing capacity of the crude extracts was measured at an absorbance of 700 nm against the methanol blank, and ferric content was estimated from the standard linear graph as mg Fe (II) equivalent per gram (g) of crude extracts.

#### 2.7.3. Assay with 2,2′-Azino-bis(3-ethylbenzothiazoline-6-sulphonic acid) (ABTS)

This was assayed following Olatunji and Afolayan (2019), with slight modifications. To produce the ABTS radical, equal volumes of 7 mM ABTS and 2.45 mM of K_2_S_2_O_8_ solutions prepared in methanol were reacted.

The resulting solution was incubated in the dark at room temperature for a duration of 12–18 h. After incubation, the mixture was diluted further to a ratio of 1:50 with methanol until the mixture’s absorbance reached 0.700 ± 0.003 when measured at 734 nm by a spectrophotometer. Afterwards, graded concentrations of water extract and standard drugs were reacted with ABTS^+^ in a ratio of 1:1 (*v*/*v*) and kept for 6 min in a dark cupboard. Immediately after incubation, absorbance was measured at 734 nm, and the percentage scavenging activities of crude extracts and standard (gallic acid), when reacted with ABTS+, were estimated as presented in Equation (2):

(2) $$\%\;\mathrm{ABTS}\;\mathrm{scavenging}\;\mathrm{activity}\;=\;[\frac{\left(\mathrm{Abs}\;\mathrm{control}\;-\;\mathrm{Abs}\;\mathrm{sample}\right)}{\left(\mathrm{Abs}\;\mathrm{control}\right)}]\;\times \;100$$

### 2.8. Statistical Analysis

All readings were obtained in triplicates. MINITAB 17 and SPSS 20 statistical packages were used to analyze the data and generate descriptive statistics of the tested samples. A one-way analysis of variance (ANOVA) was used to compare means, which were ranked using Fisher’s Least Significant Difference (LSD) pairwise comparison. Ranked means were considered significantly different at *p* < 0.05.

## 3. Results

### 3.1. Cytotoxic Effect 

**Figure 1:**
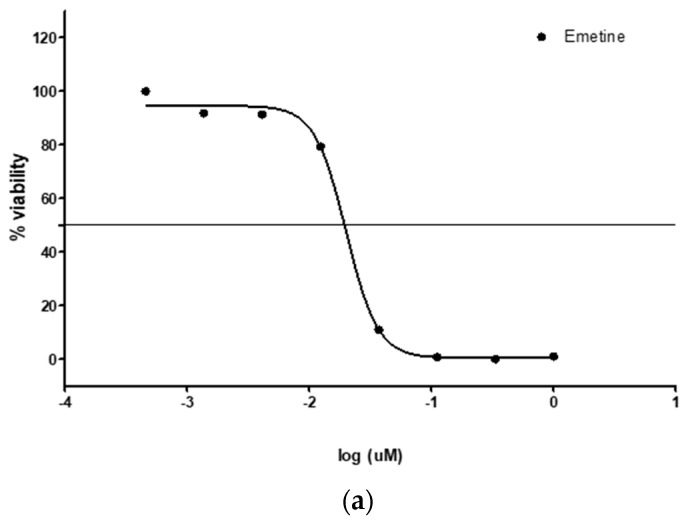

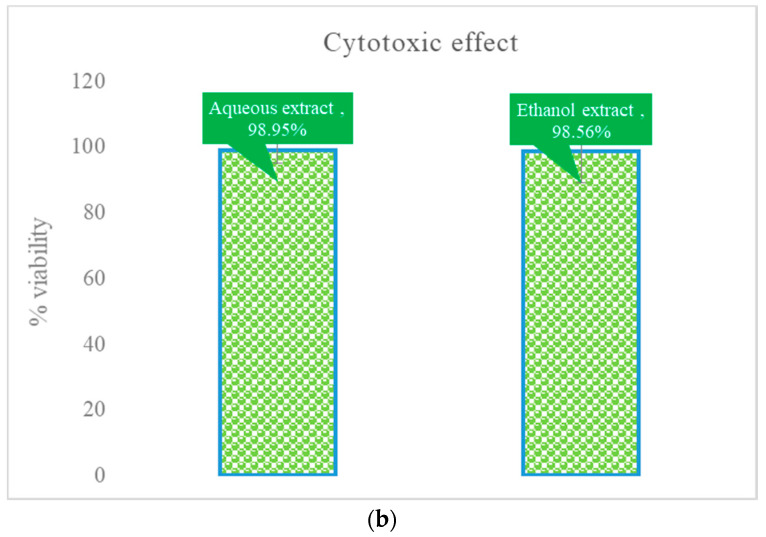
(**a**) Emetine standard @ IC_50_ – 0.020 µM; (**b**) % viability of HeLa cells with water and ethanol extracts.

The result of the cytotoxicity experiment was expressed as % viability of human cervix adenocarcinoma (HeLa) cells when treated with 50 µg/mL of crude extracts. The result showed that all extracts were not cytotoxic, as HeLa cells presented 98.95 ± 3.82 and 98.56 ± 9.54 % viability in water and ethanol extracts, respectively (Figure 1a,b). This revealed that the consumption of the velvet bean of *M. pruriens* is non-toxic and hence safe. 

### 3.2. Antimalarial (pLDH) Activity

**Figure 2:**
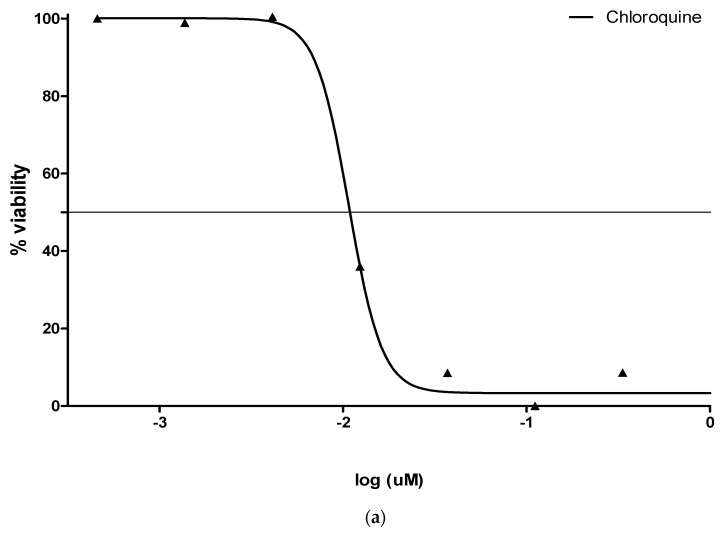

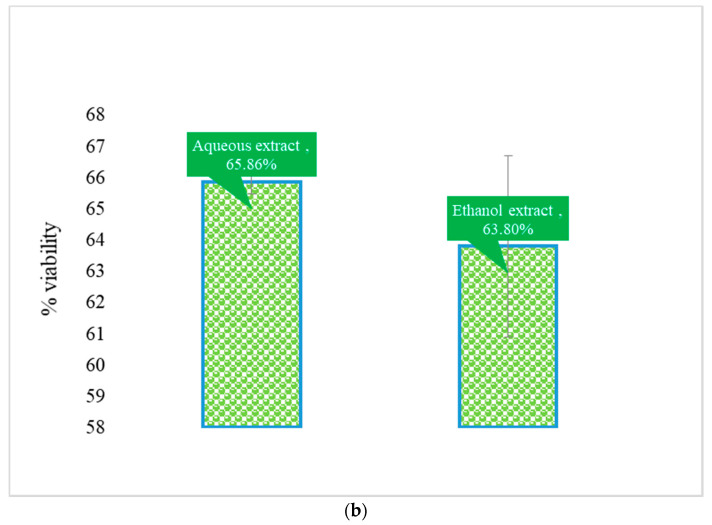
(**a**) Chloroquine @ 0.010 µM IC_50_; (**b**) % viability of *Plasmodium falciparum* strain with water and ethanol extracts.

The pLDH activity measured in compound-treated samples was expressed as % viability of *P. falciparum* strain 3D7 in a mixture of plant extracts and parasite culture relative to untreated controls. The result showed that at a fixed concentration of crude extracts (25 µg/mL), the pLDH activity showed no significant reduction by at least 50%, as the viability of the parasite ranged between 65.86 ± 0.39% and 63.80 ± 2.90 % in aqueous and ethanolic extracts, respectively. Although the tested samples showed activity, a rise in the concentration of the crude extracts might push pLDH activity below 50% (Figure 2a,b). 

### 3.3. Trypanocidal Effect

**Figure 3:**
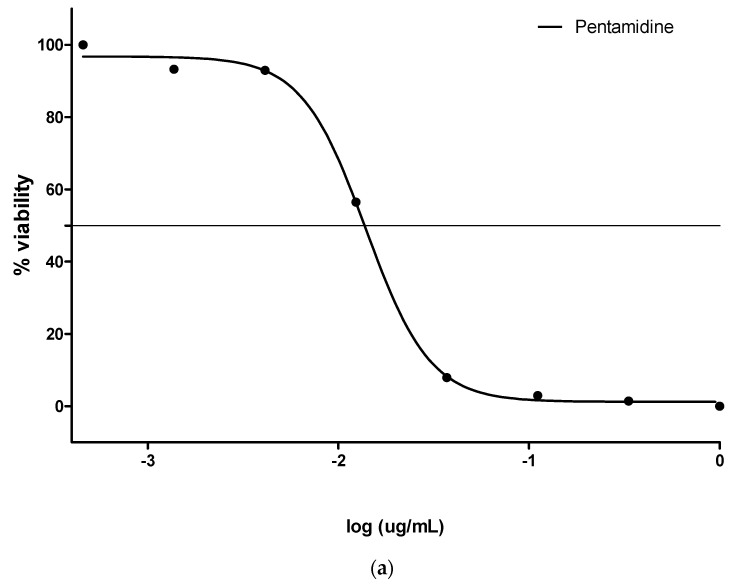

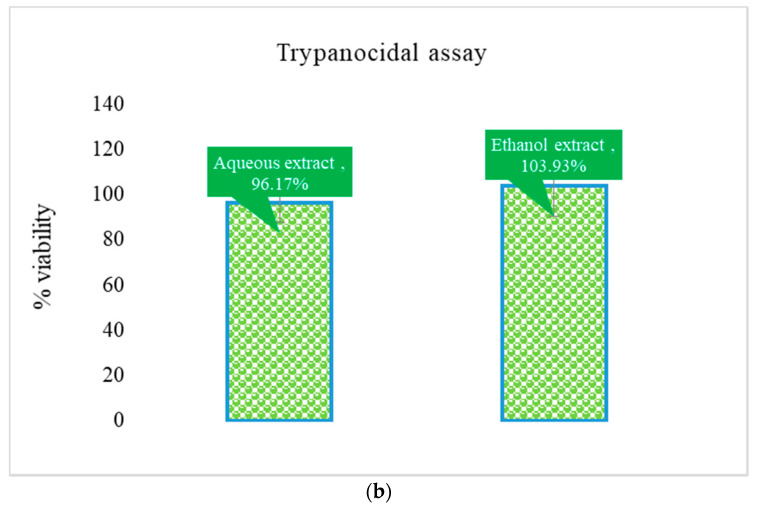
(**a**) Pentamidine standard @ IC_50_ ~ 0.014 µM; (**b**) % viability of *Trypanosoma brucei* culture with water and ethanol extracts.

The trypanocidal effect estimated in the resorufin fluorescence-tested samples was expressed as the percentage viability of a *T. brucei* culture when mixed with plant extracts relative to untreated controls (pentamidine). The result showed that at a fixed concentration of crude extracts (25 µg/mL), the parasites survived the treatment with the extracts. The viability of the parasite cultures was 96.17 ± 8.27% and 103.93 ± 13.41% in water and ethanol extracts, respectively (Figure 3a,b).

### 3.4. Phytochemical Screening

The total phenolic, flavonoid, and proanthocyanidin contents of the aqueous extract are presented in Table 1. The total phenolic content of the aqueous extract was very high compared to those of other phytochemicals (flavonoids and proanthocyanidin) evaluated. This is a strong indication that *M. pruriens* is very rich in phenols.

### 3.5. Percentage DPPH Scavenging Activity

**Figure 4:**
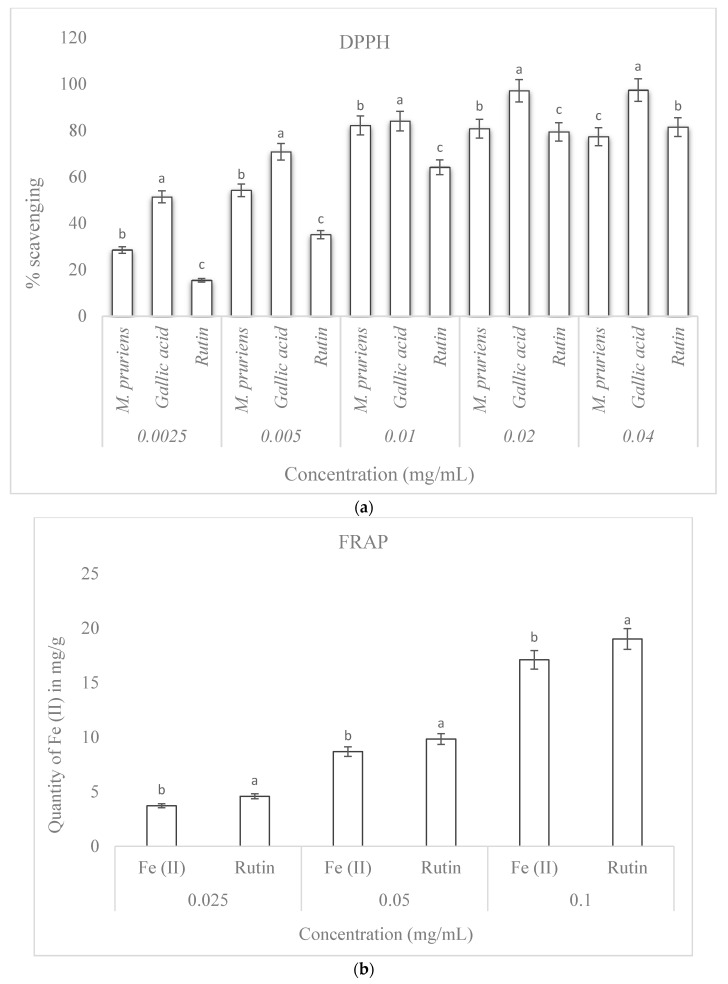

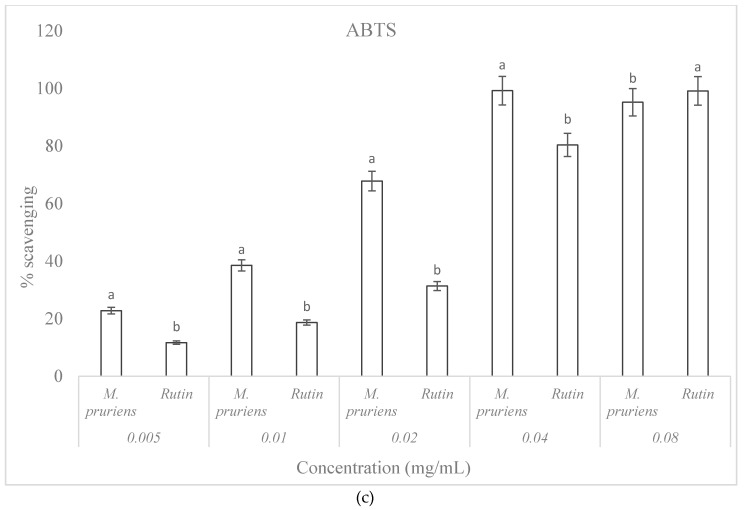
(**a**) Percentage 2,2-diphenyl-1-picrylhydrazyl eth (DPPH) scavenging activity of *Mucuna pruriens.* Mean values are compared per concentration, the values that do not share a letter are significantly different; (**b**) Ferric reducing antioxidant power of *M. pruriens.* Mean values are compared per concentration, the values that do not share a letter are significantly different; (**c**) Percentage 2,2′-azino-bis (3-ethylbenzothiazoline-6-sulphonic acid) (ABTS) free radical scavenging capacity of *M. pruriens.* Mean values are compared per concentration, the values that do not share a letter are significantly different.

The plant extract showed high scavenging activity in the DPPH free radical assay, comparable to those of the standard drugs used. At all concentrations, gallic acid showed the highest activity, while the scavenging activity of the extracts was higher than that of rutin at lower concentrations (0.0025, 0.005, 0.01, and 0.02 mg/mL) of crude extracts but lower than that of rutin at the highest concentration of crude extracts, corresponding to 0.04 mg/mL (Figure 4a).

### 3.6. Ferric Reducing Antioxidant Power

The reducing capacity of aqueous extracts of *M. pruriens* and of a standard drug estimated from the ferric (II) content showed that *M. pruriens* had a high reducing capacity although lower than that of the standard drug (rutin). The result further showed that the ferric (II) content was concentration-dependent, as it increased with a corresponding increase in the concentration (Figure 4b). 

### 3.7. ABTS Free Radical Scavenging Capacity

With ABTS radicals, the plant extracts showed higher activity at lower concentrations compared with rutin; however, rutin showed higher activity at the highest concentration (0.04 mg/mL) in a concentration-dependent manner similar to what observed for the DPPH radicals (Figure 4c).

## 4. Discussion

Plant bioactivity depends on its phytochemical richness, which in turn affects its antioxidant capacity [12]. These phytochemicals play overlapping roles in the attraction of pollinators, allelopathy, radiation absorption, singlet oxygen scavenging activity, plant defense, reduced oxidative activity, and the catalytic effect of transition metals (phytochelatins and metallothioneins) [22,23].

The polarity of solvents used for the extraction plays a crucial role in the biological activity of plants [24]. The solvents used for the extraction (water and ethanol) in this study are polar. Polar solvents are amphiprotic and can break covalent bonds by the acid–base reaction. This can facilitate the dissolution of the polar component of plant phytochemicals, such as amines, phenols, polysaccharides, ketones, and other compounds with oxygen, capable of forming hydrogen bonds [25]. Only water was used for phytochemical screening in this study, as extracts derived from the two solvents had no significant difference in cytotoxicity, trypanocidal, and antimalarial assays.

The toxicity and potential adverse effects of synthetic drugs have created fear in the mind of the people. This concern is a major driver for the patronage of natural products [26,27]. The cytotoxic treatment of human cervix adenocarcinoma (HeLa) cells with crude extracts of *M. pruriens* seeds, however, showed that the seeds were not toxic at 50 µg/mL, therefore suggesting that *M. pruriens* seeds are safe for human consumption [1,7]. Also, the plant extracts at a fixed concentration of 25 µg/mL showed a mild activity against a culture of a *P. falciparum* strain. An increase in the concentration of the crude extracts could increase the toxicity of the extracts toward *P. falciparum*. This corroborates previous folk and pharmacological reports that *M. pruriens* is a natural antimalarial remedy [3,5,15]. In this study, the high survival of *T. brucei* cultures when mixed with the plant extracts compared to viability following treatment with pentamidine, a drug administered against trypanosomiasis, is an indication that the extracts were not toxic toward *T. brucei*. This may be related to the minimal toxicity exhibited by the extracts to human cells [28,29].

Furthermore, polyphenolic compounds are pharmacological precursors and have been reported to be responsible for antioxidant activities in plants [21,30]. When interacting with legume proteins, these polyphenolic compounds exhibit enhanced antioxidant effects, higher solubility, and better emulsifying capacity [31]. Thus, the elevated phenolic content recorded for *M. pruriens* in this study might be responsible for the antioxidant activities demonstrated. Although the ABTS and DPPH radical assays share a similar mechanism of action, both were excellent tools for assessing the capacity of the crude extracts to donate labile hydrogen atoms to inhibit oxidation [32,33]. Compared with rutin and gallic acid, the stronger radical scavenging capacities of *M. pruriens*, especially at the low concentrations used in this study, agrees with early reports that the plant exhibits dose-dependent scavenging ability of hydroxyl radicals [4,6].

Additionally, the FRAP assay indicated that *M. pruriens* has a good phytochelatin content. The study revealed that the quantity of Fe (II) trapped by the aqueous extract was high and concentration-dependent, though slightly lower than the estimated quantity in the sample treated with the standard drug (rutin). At a concentration of 0.025 mg/mL of the plant and rutin samples, 3.71 and 4.58 mg/g of Fe (II) were, respectively, estimated while at 0.05 mg/mL, 8.68 and 9.84 mg/g were estimated, respectively. Also, 17.10 mg/g and 19.01 mg/g of Fe (II) were, respectively, estimated in 0.1 mg/mL of the crude extract and rutin. The output of this study further corroborates previous reports that elucidated the iron chelating capacity of *M. pruriens* [34].

## 5. Conclusion

Various pharmacological and biological activities of most natural products, particularly those derived from plants, are due to polyphenols. A high phenolic content is a major driver of the antioxidant and therapeutic activities of *M. pruriens*. On the basis of its medicinal properties, it is suggested that *M. pruriens* seeds be incorporated into human diets as a supplement to promote healthy living. This will benefit both high- and low-income earners, improving health in the former and reducing hunger and malnutrition in the latter.

## Figures and Tables

**Table 1:** Quantitative analysis of phytochemicals in aqueous extracts. GAE, gallic acid equivalent, QU, quercetin equivalent, CE, catechin equivalent.

*S*/*N*	Phytochemicals	Quantity
1	Total phenol	3730.1 ± 15.52 (mg GAE/g)
2	Flavonoids	63.03 ± 1.95 (mg QE/g)
3	Proanthocyanidins	18.92 ± 1.09 (mg CE/g)

## Abbreviations

ABTS	2,2′-Azino-bis(3-ethylbenzothiazoline-6-sulphonic acid)
CE	Catechin equivalent
FRAP	Ferric reducing antioxidant power
GAE	Gallic acid equivalent
L-DOPA	L-3,4-Dihydroxyphenylalanine
NBT	Nitro blue tetrazolium chloride solution
PES	Polyethersulfone solution
pLDH	Parasite lactate dehydrogenase
QE	Quercetin equivalent
RPMI	Roswell Park Memorial Institute
